# Efficacy and safety of fondaparinux versus enoxaparin in patients undergoing percutaneous coronary intervention treated with the glycoprotein IIb/IIIa inhibitor tirofiban

**DOI:** 10.1186/s40779-016-0081-6

**Published:** 2016-04-27

**Authors:** Xin Zhao, Xiao-Xu Yang, Su-Zhen Ji, Xiao-Zeng Wang, Li Wang, Chong-Huai Gu, Li-Li Ren, Ya-Ling Han

**Affiliations:** Cardiovascular Research Institute, Department of Cardiology, Shenyang Northern Hospital, Shenyang, Liaoning 110016 China; Department of Cardiology, Second Affiliated Hospital of Shenyang Medical College, Shenyang, Liaoning 110000 China

**Keywords:** Acute coronary syndrome, Fondaparinux, Enoxaparin, Anticoagulation, Tirofiban

## Abstract

**Background:**

In worldwide, the mortality rate of acute myocardial infarction (AMI) raises year by year. Although the applications of percutaneous coronary intervention (PCI) and anticoagulants effectively reduce the mortality of patients with acute coronary syndrome (ACS), but also increase the incidence of bleeding. Therefore, drugs with stable anticoagulant effects are urgently required.

**Methods:**

We enrolled 894 patients with acute coronary syndrome who underwent percutaneous coronary intervention in Shenyang Northern Hospital from February 2010 to May 2012; 430 patients were included in the fondaparinux group (2.5 mg/d), and 464 were included in the enoxaparin group (1 mg/kg twice daily). Fondaparinux and enoxaparin were applied for 3–7 days. All patients were treated with tirofiban (10 μg/kg for 3 min initially and 0.15 μg/(kg · min) for 1 to 3 days thereafter). The primary efficacy endpoint was the incidence of a major adverse cerebrovascular or cardiovascular event. The primary safety endpoint was bleeding within 30 days and 1 year after percutaneous coronary intervention.

**Results:**

One-year data were available for 422 patients in the fondaparinux group and for 453 in the enoxaparin group. The incidence of a major adverse cerebrovascular or cardiovascular event (10.9 % vs 12.6 %, *P* = 0.433) and cardiac mortality (0.5 % vs 1.5 %, *P* = 0.116) were generally lower in the fondaparinux group than in the enoxaparin group, although the differences were not significant. Compared with the enoxaparin group, the fondaparinux group had a significantly decreased rate of bleeding at 30 days (0.9 % vs 2.8 %) and 1 year (2.4 % vs 5.4 %). In addition, the rate of major bleeding events was lower in the fondaparinux group, but this difference was not significant (0.2 % vs 0.9 %, 0.2 % vs 1.1 %).

**Conclusions:**

In tirofiban-treated patients with acute coronary syndrome undergoing percutaneous coronary intervention, fondaparinux presented similar efficacy for ischemia events as enoxaparin. However, fondaparinux significantly decreased the incidence of bleeding, thus providing safer anticoagulation therapy.

## Background

Approximately 55 million people die each year worldwide. An epidemiological survey reports that approximately 30 % die from cardiovascular diseases, and almost 40–50 % of these deaths are caused by acute myocardial infarction (AMI) [1]. Therefore, timely and effective treatments for acute coronary syndrome (ACS) play an important role in reducing the mortality of patients with ACS. ACS is mainly due to the erosion of the fibrous cap on the coronary artery atheromatous surface. After this erosion, platelets adhere and aggregate to the ruptured surface. Ultimately, thrombosis occurs due to activation of the blood coagulation cascade. According to the degree of arterial obstruction, ACS is divided into unstable angina, ST segment elevation myocardial infarction (STEMI), and non-ST segment elevation myocardial infarction (NSTEMI).

As anticoagulants, unfractionated heparin (UFH) and low-molecular-weight heparin (LMWH) have successfully been used to reduce the incidence of ischemic complications in patients with ACS undergoing percutaneous coronary intervention (PCI); however, these agents also increase the incidence of bleeding [2, 3]. Patients with ACS have serious coronary insufficiency, and the anticoagulant activity is unpredictable. Therefore, drugs with stable anticoagulant effects are urgently required. Platelet activation and aggregation play crucial roles in the blood coagulation cascade, which causes MI events and thrombotic complications in the perioperative period of PCI. Previously obtained data have shown that the inhibition of platelet activity that results from the application of glycoprotein IIb/IIIa inhibitors (GPIs) during PCI is effective for protection against ischemic events [4]. GPIs act by inhibiting fibrinogen and von Willebrand factor, which mediate platelet cross-linkage, the final stage of platelet aggregation. In Europe and the United States, GPIs are frequently used in patients with STEMI who are undergoing PCI to reduce the incidence of ischemic complications [5–7]. However, the application of antiplatelet therapy and thrombolytic therapy during PCI can apparently increase the incidence of hemorrhagic events and thrombocytopenia [8–11], and major bleeding can predict myocardial infarction, stroke and death [12]. Consequently, although antiplatelet and thrombolytic therapies for ACS have the benefit of restoring perfusion by preventing further platelet aggregation and thrombotic formation, they also reduce the risk of ischemic events; however, this comes at the cost of a higher risk of bleeding. Therefore, clinical studies are attempting to determine how to maximize the benefit while minimizing the risk associated with anticoagulant therapy.

The synthetic inhibitor of coagulation factor X, fondaparinux, (Arixtra; GlaxoSmithKline) has been recently evaluated in patients with ACS who were enrolled in the Fifth and Sixth Organization to Assess Strategies in Ischemic Syndromes (OASIS 5 and 6) trials [13, 14]. In these trials, patients were treated using conservative or invasive strategies. The OASIS-5 trial demonstrated that in >20,000 patients with NSTEMI, fondaparinux reduced major bleeding by 50 % and reduced mortality by 17 % at 9 days compared with enoxaparin [13]. The anticoagulant enoxaparin is superior to UFH in reducing the risk of MI or death [15]. Although the beneficial effects of fondaparinux at 9 and 30 days are known, the results at 1 year remain unknown. The objective of the present study was to detect the efficacy and safety of fondaparinux and enoxaparin in patients with ACS undergoing PCI when treated with GPI tirofiban.

## Methods

### Participants

In this study, we collected information regarding patients with ACS who underwent PCI in Shenyang Northern Hospital from February 2010 to May 2012; all patients were treated with tirofiban, and 894 patients were enrolled. Of these, 430 patients were enrolled in the fondaparinux (2.5 mg/d) group, and 464 were enrolled in the enoxaparin group (1 mg/kg twice daily) for the index procedure. Fondaparinux and enoxaparin were administered for 3–7 days according to the coronary artery lesions during PCI. All patients were treated with tirofiban (10 μg/kg for 3 min initially and 0.15 μg/(kg · min) for 1 to 3 days thereafter). All patients and their relatives provided informed consent, and the study was approved by the ethical committee of Shenyang Northern Hospital.

Patients aged ≥18 years were eligible for enrollment if they exhibited ischemic symptoms within 24 h and met the following criteria: (1) elevated creatine kinase–myocardial band (CK-MB) isoenzyme or troponin, or an electrocardiographic change indicating ischemia; (2) planned elective PCI; (3) treated with GPI tirofiban. The patients were also treated with enteric-coated aspirin, clopidogrel, nitrates, β-blockers, angiotensin-converting enzyme inhibitors (ACEI) or angiotensin receptor blockers (ARBs) and statins.

The following exclusion criteria were applied: planned to undergo emergency PCI in patients with ACS; treated with thrombolysis within 3 days of STEMI; left main coronary artery disease; cardiogenic shock with a high possibility of vascular lesions or secondary bleeding, such as gastric ulcer or intracranial aneurysm; planned to undergo coronary artery bypass surgery; previous treatment with fibrinolytic therapy, such as bivalirudin, GPI, LMWH, or fondaparinux (UFH was allowed); current application of Coumadin; stroke or transient ischemic attack within the past 6 months; digestive tract or urinary tract hemorrhage within the past 3 months; cerebral infarction within the past 3 months and cerebral hemorrhage within the past 6 months; platelet count <100,000 cells/μl or hemoglobin concentration <100 g/L; planned elective surgical procedure that would necessitate anticoagulation interruption; coronary stent implantation within the past 30 days; abnormal liver function (alanine aminotransferase or aspartate aminotransferase 1.5 times normal value) or renal impairment (serum creatinine level >180 mmol/L).

### Outcome

The primary efficacy endpoint was the occurrence of major adverse cerebrovascular or cardiovascular events (MACCEs), which were defined as comprising cardiac mortality, non-fatal MI, emergent target vessel revascularization (TVR), and ischemic stroke at 1 year. The primary safety endpoints were bleeding events within 30 days and 1 year after index PCI and were evaluated using the thrombolysis in myocardial infarction (TIMI) definition. MI was defined as follows: prolonged ischemic symptoms with CK-MB isoenzyme levels more than three times the upper limit of normal, electrocardiographic changes indicating ischemia or the appearance of new Q waves. TVR included intervention due to the recurrence of any part of the original vessel. Bleeding events were as defined by the TIMI test group and were divided into major, secondary and minor bleeding [16].

### Statistical analysis

The data were analyzed using SPSS version 19.0. Baseline characteristics were represented as means ± SD or medians (range) and were compared using the *t* test; proportions were compared using the *χ*^2^ test. Fisher’s test was used to compare exact probability. A Cox proportional hazards model was used to analyze MACCEs and all bleeding events in the fondaparinux and enoxaparin groups. The Cox proportional hazards model was used to calculate the odds ratios for the morbidity of MACCEs in the two groups. All *P* values were two-tailed, and statistical significance was defined as *P* < 0.05.

## Results

**Fig. 1:**
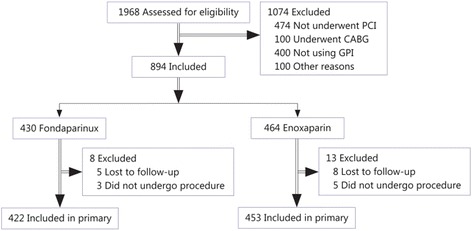
Trial profile. Abbreviations: PCI, percutaneous coronary intervention; CABG, Coronary Artery Bypass Grafting; GPI, glycoprotein IIb/IIIa inhibitor

As described above, there were 430 patients in the fondaparinux group and 464 in the enoxaparin group at enrollment. Follow-up data were available for 422 (98.1 %) patients in the fondaparinux group and 453 (97.8 %) in the enoxaparin group. Reasons for dropout were loss to follow-up or withdrawal of consent (Fig. 1).

The baseline clinical characteristics of sex, age, weight, operative condition for PCI, and application of tirofiban did not significantly differ between the two groups (*P* > 0.05); in addition, the application period of fondaparinux and enoxaparin did not significantly differ between the two groups (4.75 ± 1.81 vs 4.57 ± 1.75, *P =* 0.168). However, the proportion of patients with STEMI was higher in the fondaparinux group than that in the enoxaparin group (37.7 % vs 25.6 %, *P* < 0.001, Table 1). Administration of enteric-coated aspirin, clopidogrel, ACE inhibitors, ARBs, calcium channel blockers, β-blockers, and statins during the follow-up period did not differ significantly between the two groups (*P* > 0.05, Table 2). After 1 year of follow-up, the rate of MACCEs was lower in the fondaparinux group than in the enoxaparin group (10.9 % vs 12.6 %, *P* = 0.433), although the difference was not marked. The incidence of cardiac mortality, nonfatal MI, TVR, and stroke did not differ significantly between the fondaparinux and enoxaparin groups (*P* > 0.05). The rate of all bleeding events was significantly lower in the fondaparinux group than in the enoxaparin group at 30 days (0.9 % vs 2.8 %, *P* = 0.040) and at 1 year (2.4 % vs 5.4 %, *P* = 0.018). Although the incidence of major bleeding events was lower in the fondaparinux group than in the enoxaparin group at 30 days (0.23 % vs 0.88 %, *P* = 0.429) and at 1 year (0.23 % vs 1.1 %, *P* = 0.253), the difference was not significant (Table 3).

**Table 1:** Baseline characteristics of the fondaparinux and enoxaparin groups

Characteristics	Fondaparinux (*n* = 430)	Enoxaparin (*n* = 463)	*P*
Age (year)	57.29 ± 9.39	58.16 ± 9.58	0.170
Male [*n*(%)]	328 (76.3)	363 (78.2)	0.486
Weight (kg)	72.04 ± 12.08	72.64 ± 13.05	0.487
Hypertension [*n*(%)]	245 (57.2)	276 (59.5)	0.448
Diabetes [*n*(%)]	98 (22.8)	94 (20.3)	0.357
Stroke [*n*(%)]	14 (3.3)	11 (2.4)	0.423
Prior myocardial infarction [*n*(%)]	50 (11.6)	74 (15.9)	0.060
Current smoker [*n*(%)]	224 (52.1)	246 (53.0)	0.782
ACS [*n*(%)]			
Unstable angina	216 (50.2)	296 (68.3)	0.658
Non-ST-elevation ACS	52 (12.1)	49 (10.6)	0.477
ST-elevation MI	162(37.7)	119 (25.6)	<0.001
Target vessel position [*n*(%)]			
Left Main	53 (12.3)	66 (14.2)	0.397
Left Anterior Descending	278 (64.7)	295 (63.6)	0.771
Left Circumflex	36 (8.4)	39 (8.4)	0.978
Right Coronary Artery	46 (10.9)	45 (9.7)	0.629
PCI			
Average stent diameter (mm)	3.03 ± 0.40	3.06 ± 0.40	0.181
Average length of stents (mm)	27.17 ± 5.96	27.58 ± 5.89	0.293
Average number of stents	1.85 ± 0.96	1.89 ± 1.02	0.627
Transradial artery invasive [*n*(%)]	378 (87.9)	408 (87.9)	0.991
Tirofiban (d)	1.84 ± 0.66	1.90 ± 0.62	0.495
Fondaparinux/enoxaparin (d)	4.75 ± 1.81	4.57 ± 1.75	0.168
Laboratory examination			
APTT (s)	33.39 ± 5.86	32.97 ± 5.57	0.271
Prothrombin time (s)	12.97 ± 1.38	12.90 ± 1.07	0.381
HDL-C (mmol/L)	1.19 ± 0.44	1.18 ± 0.58	0.639
Creatinine (mmol/L)	87.50 ± 18.71	91.02 ± 15.02	0.116
PLT (×10^9^/L)	212.29 ± 56.09	212.50 ± 51.13	0.954

*ACS* Acute coronary syndrome, *MI* Myocardial infarction, *PCI* Percutaneous coronary intervention, *APTT* Activated partial thromboplastin time, *HDL-C* High-density lipoprotein cholesterol. All values are expressed as means ± SD. *P* < 0.05 was considered to indicate statistical significance

**Table 2:** Application of drugs during follow-up

Medicine	Fondaparinux (*n* = 422)	Enoxaparin (*n* = 453)	*P*
Aspirin [*n*(%)]	419(99.3)	452(99.8)	0.567
Clopidogrel [*n*(%)]	338(80.1)	341(75.3)	0.088
ACE inhibitor or ARB [*n*(%)]	353(83.6)	379(83.7)	0.995
Calcium-channel blocker [*n*(%)]	69(16.4)	72(15.9)	0.854
Beta-blocker [*n*(%)]	360(85.3)	398(87.9)	0.268
Statin [*n*(%)]	397(94.0)	425(93.8)	0.874

*P* < 0.05 was considered to indicate statistical significance

**Table 3:** Clinical outcomes at one year in patients undergoing PCI [*n*(%)]

Variables	Fondaparinux (*n* = 422)	Enoxaparin (*n* = 453)	*P*
MACCE^a^	46 (10.9)	57 (12.6)	0.433
Death or MI	18 (4.3)	24 (5.3)	0.475
Death	2 (0.5)	7 (1.5)	0.116
MI	16 (3.8)	17 (3.8)	0.976
TVR	23 (5.5)	28 (6.2)	0.645
Stroke	7 (1.7)	9 (2.0)	0.717
30 day			
Major bleeding	1 (0.23)	4 (0.88)	0.429
All bleeding^b^	4 (0.95)	13 (2.9)	0.040
1 year			
Major bleeding	1 (0.23)	5 (1.1)	0.253
All bleeding^b^	10 (2.4)	25 (5.5)	0.018

*MI* Myocardial infarction, *TVR* Target vessel revascularization. ^a^MACCE: including death from cardiovascular causes, non-fatal myocardial infarction and target vessel revascularization. ^b^All bleeding was defined using the TIMI definition. *P* < 0.05 was considered to indicate statistical significance

**Fig. 2:**
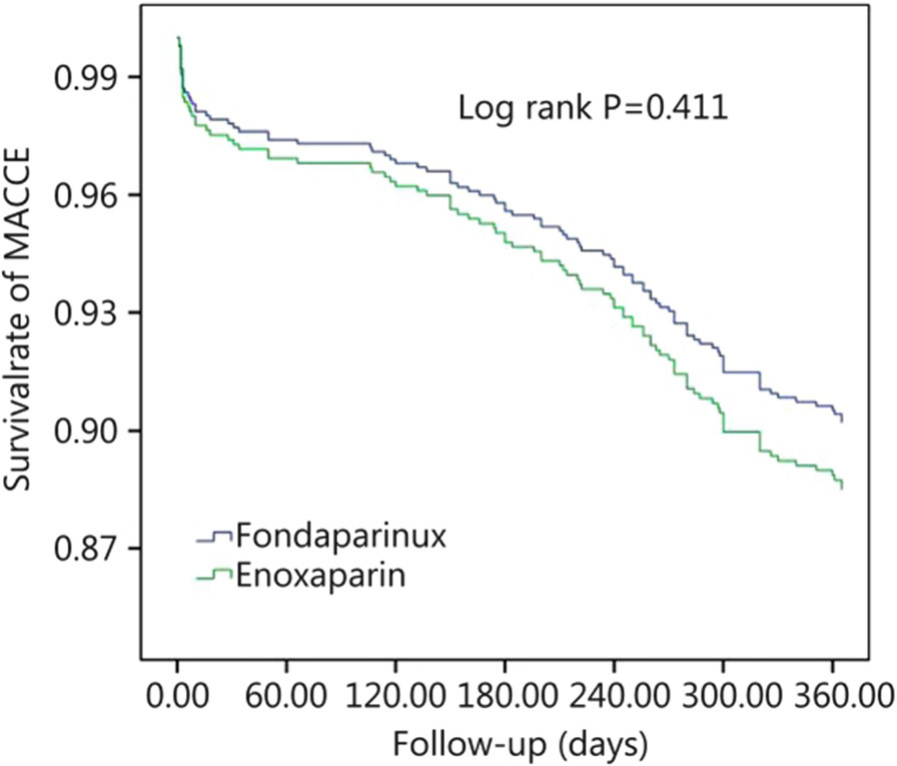
Efficacy of fondaparinux and enoxaparin in patients undergoing percutaneous coronary intervention. MACCE, including death from cardiovascular causes, non-fatal myocardial infarction and target vessel revascularization. *P* < 0.05 was considered to indicate statistical significance

**Fig. 3:**
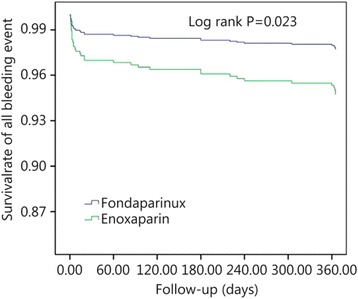
Safety of fondaparinux and enoxaparin in patients undergoing percutaneous coronary intervention. *P* < 0.05 was considered to indicate statistical significance

Figure 2 shows the efficacy and safety of fondaparinux and enoxaparin in patients undergoing PCI. After adjusting for age, sex, and administration of antiplatelet drugs, the incidence rate of all bleeding events was significantly lower in the fondaparinux group than the enoxaparin group at 1 year of follow-up, according to the concomitant use of GPI (*P* = 0.023). After adjusting for age, sex, weight and other cardiovascular disease risk factors, fondaparinux did not increase the risk of MACCEs compared with enoxaparin (*P* = 0.411, Fig. 3).

We also evaluated the incremental risk factors in these patients at high risk for MACCEs and found that history of diabetes was an independent risk factor for the incidence of MACCEs. Male sex, age, prior PCI, and other factors were not significantly associated with MACCE incidence (Table 4).

**Table 4:** Odds ratios for the morbidity of MACCE

Variable	*OR*	95 % CI	*P*
Male	0.784	0.454–1.356	0.384
Age	1.017	0.994–1.041	0.155
ACS	1.024	0.806–1.300	0.848
Diabetes	2.028	1.298–3.169	0.002
Creatinine	0.991	0.977–1.005	0.206
Prior PCI	1.424	0.821–2.471	0.208
Prior myocardial infarction	1.126	00619–2.047	0.697
NYHA functional classification	1.035	0.663–1.614	0.881

*ACS* Acute coronary syndrome. *P* < 0.05 was considered to indicate statistical significance

## Discussion

Currently, multiple agents are used in clinical work [17]. The application of thrombin inhibitors and invasive treatments can substantially reduce the occurrence of ischemic events but can also increase the incidence of bleeding in patients with ACS. Among these antiplatelet agents, GPIs exhibit a significant benefit regarding the occurrence of MI events and mortality in patients with ACS; however, GPIs also lead to a 60 % increase in bleeding (*OR* = 1.62, 95 % CI: 1.36–1.94) [18]. Several studies have found that GPIs can increase the occurrence of MI events, stroke and death in patients with bleeding events [12, 19]. The mechanisms behind the increased mortality or morbidity associated with bleeding are unclear, but may be associated with fatal bleeding, hemorrhagic shock, hypoxia (possibly of particular relevance in STEMI patients), cessation of therapy (such as β-blockers and ACE inhibitors), systemic inflammation, adverse effects of transfusion and visceral dysfunction [20–23]. Because of the serious long-term consequences of major bleeding, treatment strategies that can reduce the risk of bleeding while maintaining the benefits of decreasing ischemic events are required. In our study, we found that fondaparinux significantly decreased the incidence of bleeding events in patients undergoing PCI with GPI, representing an alternative to enoxaparin that preserves efficacy while improving safety [13].

In the OASIS-5 trial, fondaparinux reduced major bleeding by 48 % at 9 days in patients undergoing PCI with non-ST-segment elevation ACS, which was associated with a 17 % mortality reduction at 30 days [13]. However, the rate of catheter-related thrombosis was higher in those treated with fondaparinux than with enoxaparin (0.9 % vs 0.4 %, respectively), which limited the widespread adoption of fondaparinux for patients with ACS. In the FUTURA/OASIS-8 trial, we found a higher rate of guiding catheter-related thrombosis in patients who had undergone PCI that was performed without UFH; however, this higher rate was largely avoided when UFH was used before the procedure [24].

In the present study, we found that the incidence of MACCEs and cardiac mortality was lower in the fondaparinux group than in the enoxaparin group. Furthermore, the incidence of all bleeding events was significantly lower in the fondaparinux group than in the enoxaparin group. We also evaluated incremental risk factors in patients at a high risk of MACCEs and found that diabetes was an independent risk factor of MACCEs in patients with ACS.

In the OASIS-6 trial, the incidence of major bleeding events was not increased by the application of fondaparinux; furthermore, cardiac tamponade was clearly reduced, and a trend towards fewer fatal bleeding events was observed [14]. The trend toward lower rates of fatal and life-threatening bleeding was consistent with the markedly lower rates of bleeding in the fondaparinux group compared with the enoxaparin group found in the OASIS-5 trial. This finding suggests that fondaparinux might decrease the risk of major bleeding at the doses used. In our present trial, we found that fondaparinux significantly reduced the rate of bleeding and resulted in a lower incidence of MACCEs compared with enoxaparin. One patient in the enoxaparin group exhibited gastrointestinal bleeding (major bleeding as defined by TIMI) at 1 day after PCI; antithrombotic therapy and aspirin were discontinued, and the dose of clopidogrel was changed to 150 mg/day. However, after 4 days, the patient died from cardiogenic shock and organ failure. We consider that, in addition to cardiogenic shock and liver and kidney dysfunction in patients with AMI, the cessation of antithrombotic therapy and decreased oxygen delivery have adverse effects on prognosis. Further study is needed to elucidate the association of bleeding and ischemic events, and the specific mechanisms involved.

Compared with LMWH, fondaparinux was found to specifically inhibit and reduce the production of factor Xa but did not completely prevent the formation of thrombin. This drug also prevented excessive anticoagulation, a finding that partly explains why fondaparinux has a stable anticoagulant effect and does not increase bleeding in patients with ACS. Particularly in patients with advanced age, diabetes mellitus or renal insufficiency, fondaparinux has a half-life of 15 h, obviating the need for laboratory monitoring; thus, fondaparinux can be recommended.

Our study had the following limitations. First, the follow-up was short and the sample was small; therefore, longer follow-up and larger sample size are needed. Second, the study was retrospective, which inevitably resulted in recall bias.

## Conclusions

In summary, in patients with ACS treated with GPI tirofiban undergoing PCI, the efficacy of fondaparinux is not inferior to that of enoxaparin. However, fondaparinux could decrease the incidence of bleeding after PCI; therefore, fondaparinux treatment for ACS is safe and can be recommended.

## Abbreviations

AMI: acute myocardial infarction
ACS: acute coronary syndrome
ACEI: angiotensin-converting enzyme inhibitors
ARBs: angiotensin receptor blockers
CK-MB: creatine kinase–myocardial band
GPIs: glycoprotein IIb/IIIa inhibitors
LWMH: low-molecular-weight heparin
MACCEs: major adverse cerebrovascular or cardiovascular events
NSTEMI: non-ST segment elevation myocardial infarction
PCI: percutaneous coronary intervention
STEMI: ST segment elevation myocardial infarction
TVR: target vessel revascularization
TIMI: thrombolysis in myocardial infarction
UFH: unfractionated heparin
